# The interconnection and function of associative memory neurons are upregulated for memory strengthening

**DOI:** 10.3389/fncir.2023.1189907

**Published:** 2023-06-15

**Authors:** Jia-Yi Li, Yang Xu, Dan-Gui Wang, Jin-Hui Wang

**Affiliations:** College of Life Science, University of Chinese Academy of Sciences, Beijing, China

**Keywords:** associative learning, barrel cortex, synapse, neurons, axon

## Abstract

Memories associated to signals have been proven to rely on the recruitment of associative memory neurons that are featured by mutual synapse innervations among cross-modal cortices. Whether the consolidation of associative memory is endorsed by the upregulation of associative memory neurons in an intramodal cortex remains to be examined. The function and interconnection of associative memory neurons were investigated by *in vivo* electrophysiology and adeno-associated virus-mediated neural tracing in those mice that experienced associative learning by pairing the whisker tactile signal and the olfactory signal. Our results show that odorant-induced whisker motion as a type of associative memory is coupled with the enhancement of whisking-induced whisker motion. In addition to some barrel cortical neurons encoding both whisker and olfactory signals, i.e., their recruitment as associative memory neurons, the synapse interconnection and spike-encoding capacity of associative memory neurons within the barrel cortex are upregulated. These upregulated alternations were partially observed in the activity-induced sensitization. In summary, associative memory is mechanistically based on the recruitment of associative memory neurons and the upregulation of their interactions in intramodal cortices.

## Introduction

Associative learning and memory as the most common style of information acquisition are believed to be essential for cognitive activities and emotional responses (Byrne, 1987; Wasserman and Miller, 1997; Kandel and Pittenger, 1999; Silva et al., 2009; Poo et al., 2016; Wang, 2019). Synaptic and neuronal plasticity has been presumed to be the cellular mechanism underlying learning and memory (Bliss and Lomo, 1973; Bliss and Lynch, 1988; Wang et al., 1997; Armano et al., 2000; Zhang et al., 2004; Lisman et al., 2018; Josselyn and Tonegawa, 2020). Although the neural plasticity expressed in a single synapse pathway affects the storage consolidation and the retrieval efficiency of the memorized signals within the intramodal cortices, e.g., the sensitization, this activity-dependent plasticity in the single pathway cannot be used to interpret the formation of associative memory, i.e., the joint storage and the reciprocal retrieval of those associated signals inputted from multiple pathways into cross-modal cortices (Wang, 2019). Recent studies have identified the recruitment of associative memory neurons after associative learning experiences and associative memory formation. Associative memory cells are recruited from the coactivity of neurons among cross-modal cortices based on a rule of the activity together and the interconnection together (Wang, 2019). Associative memory neurons are characterized by the reception of new synapse innervations from coactive neurons, the formation of their synapse interconnections, and the functional encoding of synapse signals received from cross-modal cortices after memories to the associated signals have formed (Wang et al., 2015; Gao et al., 2016, 2019; Yan et al., 2016; Feng et al., 2017; Lei et al., 2017; Liu et al., 2017; Wu et al., 2020). However, the refinement of associative memory neurons within the intramodal cortex for a functional modality, e.g., the upregulation of their interconnection to strengthen their interaction and excitatory state for facilitating memory retrievals, remains to be addressed *in vivo*.

After the memory formation, the associated signals can be retrieved normally by low-intensity cues and even spontaneously (Jutras and Buffalo, 2010; Wang, 2019). If a group of associative memory neurons is recruited to encode specific associated signals in the formation of associative memory, their synapse inputs and excitability are presumably upregulated to facilitate the retrieval of such associated signals (Guo et al., 2017; Zhao et al., 2017). In this regard, we expect to see the upregulations in the synapse interconnection among local associative memory neurons, the response of associative memory neurons to their coded signals, and the dominantly spontaneous activity of associative memory neurons *in vivo*. In the present study, we intend to test whether the upregulated refinement of associative memory neurons works for strengthening the storage and retrieval of the associated signals after associative learning, in addition to the potentiation of cortical neurons for non-associative learning, such as sensitization.

Strategies in our study are given below. In a mouse model of associative learning by pairing whisker and odorant stimulations, the formation of associative memory in mice was featured by the odorant-induced whisker motion alongside the innate whisking-induced whisker motion (Wang et al., 2013, 2015; Gao et al., 2016, 2019). In addition to this associative learning and memory in an experimental group of mice, other two groups of control mice included the naïve control and the control of the unpaired whisker stimulation to reveal the effects of the repetitive unpaired stimulations on innate reflex behavior and neuronal units in the barrel cortex. The interconnections among associative memory neurons were studied by injecting adeno-associated viruses that carried a gene for encoding a fluorescent protein in the cortical area with the recruitment of associative memory neurons and subsequently by detecting the synapse contacts between fluorescent-expressed axonal boutons and post-synaptic neuronal spines in this local area (Feng et al., 2017; Lei et al., 2017; Gao et al., 2019). The activity strength of the associative memory neurons was quantified by recording their sequential action potentials (i.e., spikes) in response to the learning cues and spontaneously as well (Feng et al., 2017; Wu et al., 2020).

## Materials and methods

Experiments were done in accordance with guidelines and regulations by the Administration Office of Laboratory Animals in Beijing, China. All of the experimental protocols were approved by Institutional Animal Care and Use Committee in the Administration Office of Laboratory Animals in Beijing, China (B10831).

C57BL/6J Thy1-YFP mice (Jackson Laboratory, Bar Harbor, ME, USA) were used in all our experiments, whose glutamatergic pyramidal neurons in the brain are genetically labeled by the yellow fluorescent protein (YFP) (Feng et al., 2000; Zhang et al., 2013). The mice were accommodated in sterile barrier facilities under conditions of 12-h day/night, with sufficient availability of food and water. Mice postnatal 20 days with well-developed body weight were selected for starting the training paradigm of associative learning and as controls. The timeline of our experiments was executed in the following stages, the assignation of these mice randomly into the naïve control group (NCG), unpaired-stimulus group (UPSG), and paired-stimulus group (PSG); the microinjection of AAVs into their barrel cortices, the uses of the training paradigms in the mice for 2 weeks after this surgical operation of AAV microinjections were done at around 48 h. The maintenance of these mice in their habitations at 5 days, as were the studies in behavioral tasks, morphological identifications, and electrophysiological recordings.

The paradigm of associative learning has been described in our previous studies (Wang et al., 2015; Yan et al., 2016; Liu et al., 2017; Wang, 2019). Briefly, the mice were taken into the laboratory for them to be familiar with experiment operators and the training apparatus for 2 days. A paradigm of associative learning was the pairing of whisker and odor stimulations simultaneously in these mice, which was called a paired stimulus group (PSG), in comparison with the naïve control group (NCG) and the unpaired stimulus group (UPSG). In the UPSG mice, the unpaired stimulations of the whisker signal and the odor signal were given separately to the mice with the intervals being about 5 min. On the other hand, the mice in the naïve control group did not receive either the whisker stimulus or the odorant stimulus until test experiments were given. The whisker stimulus signal was mechanical stimulations (5 Hz) to mouse longer whiskers for 20 s, which were the contralateral side of the barrel cortex with the allocation of the recruited associative memory neurons in the following study. The odor stimulus signal was a butyl acetate pulse closely to the mouse noses for 20 s. The intensity of the whisker stimulus was sufficient to trigger whisker fluctuation or an innate whisking-induced whisker motion. The odor stimulus was given by switching on the butyl acetate-containing tube to generate a small liquid drop in front of the animals’ noses. The intensity of butyl acetate was enough to activate the olfactory bulb neurons, which has been confirmed by the two-photon cell imaging (Wang et al., 2015). Temporal parameters for the whisker stimulus and the odor stimulus to the PSG mice with the paired stimulations and to the UPSG mice with the unpaired stimulations were 20 s and five times per day with 2-h intervals for 12 days. The use of this paradigm for associative learning was based on the fact that the onsets of odorant-induced whisker motion and whisking-induced olfactory response reached their plateau level by training for approximately 10 days (Wang et al., 2015; Liu et al., 2017). The intensity, duration, and frequency of whisker and odorant stimulations were controlled by a multiple sensory modal stimulator (MSMS) with the locked parameters for all mice.

The odorant-induced whisker motion was measured to identify the formation of associative memory (Gao et al., 2016, 2019; Feng et al., 2017; Lei et al., 2017; Wu et al., 2020). To quantify the onset time and strength of odorant-induced whisker motion, the mouse whisker motions in response to the testing odorant (butyl acetate for 20 s) were recorded by a digital video camera (SONY, HDR-AS100V, 240 fps) after the training. The odorant-induced whisker motion, or associative memory, was accepted when whisker motions met the following criteria. The pattern of odorant-induced whisker motion was similar to the typical innate whisker motion induced by the whisker stimulus but differed from spontaneous low-magnitude whisking. The whisking frequency and angle increased significantly, compared to those in baseline controls and control groups. This odorant-induced whisker motion was originally induced by the whisker stimulus, such that the odor signal evoked the recall of the whisker signal and then the whisker motions similar to the innate reflex (Wang et al., 2015; Liu et al., 2017; Wang, 2019). The whisker motion in response to the whisker stimulus, or an innate reflex of whisking-induced whisker motion, was also monitored by those approaches above to examine the plasticity of this innate reflex after the formation of associative memory and the type of non-associative memory, such as sensitization.

Strategies to examine whether and how the basic units in memory traces interconnect and interact each other were to identify their connection by using an AAV-carried fluorescent protein in those PSG mice that had experienced associative learning and formed associative memory, in comparison with UPSG mice and NCG mice (Gao et al., 2016, 2019; Feng et al., 2017; Lei et al., 2017; Wu et al., 2020). In our experiments, the AAV-CMV-TdTomato was microinjected into the barrel cortices by using the glass electrode before the training paradigm. The microinjections were controlled by a microsyringe held on the three-dimensional stereotaxic apparatus (RWD Life Science, Shenzhen, China). Microinjection sites in the barrel cortices were 1.7 mm posterior to the bregma, 2.75 mm lateral to the midline, and 0.7 mm in the depth (Paxinos and Watson, 2005). The quantity of injected AAVs was 0.5 μl with an injection period of about 30 min. Theoretically and practically, AAV-CMV-TdTomato was uptaken by and then expressed in cortical neurons, where red fluorescent protein (RFP) was produced. The RFP was transported toward entire axonal arbors in an anterograde manner so that axonal boutons and terminals were labeled by the RFP (Gao et al., 2016, 2019; Feng et al., 2017; Lei et al., 2017; Wu et al., 2020). AAVs were microinjected into the barrel cortex 2 days before the training paradigm for the transportation of AAV-carried genes to express RFP with the following consideration. (1) The experimental situation for the mice to experience the training process was less influenced by the injury of the surgical operation. (2) Based on the activity-dependent growth of neuronal processes, the learning-driven activities of cortical neurons make their processes to be growth. If the AAV-carried genes for encoding fluorescent proteins have been in the neurons prior to their intensive activities, they transport following the growth of neuronal axons toward their terminals for the expression. The transportation of the fluorescent proteins expressed in somata toward axon terminals as well as the transportation of AAV-carried genes toward axon terminals for their expression facilitate the accumulation of fluorescent proteins in axon boutons and terminals for the axonal labeling.

The PSG, UPSG, and NCG mice whose barrel cortices had been microinjected with AAVs experienced the training paradigms designed for each of the three groups. Three weeks after these manipulations, these mice were anesthetized by intraperitoneal injections of 4% chloral hydrate (0.1 ml/10 g) and perfused through the left ventricle with 50 ml 0.9% saline followed by 50 ml of 4% paraformaldehyde until their bodies became rigid. The brains were quickly isolated and post-fixed in 4% paraformaldehyde for additional 24 h. The cerebral brains were sliced by a vibratome in a series of coronal sections with a thickness of 100 μm. In order to clearly show three-dimensional images of new synapses in the barrel cortex, we have placed brain slices into Sca/eA2 solution for 10 min to make them transparent (Hama et al., 2011; Gao et al., 2019). These slices were rinsed with the phosphate buffer solution (PBS) three times, air-dried, and cover-slipped. Neurons, dendrites, dendritic spines, and axon boutons in layers II∼III of the barrel cortex were imaged and collected at a 60X lens for high magnification in a confocal microscope (Nikon A1R plus). Anatomic images of the cerebral brain were taken by a 4X lens for low magnification in this confocal microscope. In C57BL/6J Thy1-YFP mice, post-synaptic neuron dendrites and spines were labeled by the YFP. The presynaptic axon boutons, whose diameters were larger than the diameter of axons and less than one micrometer (Gao et al., 2016, 2019; Feng et al., 2017; Lei et al., 2017), were labeled by the RFP produced from the injected AAV-CMV-TdTomato. Those contacts between red axonal boutons and yellow dendritic spines with less than 0.1 μm space cleft were presumably chemical synapses (Gao et al., 2019; Wu et al., 2020). The wavelength of an excitation laser beam 488 nm was used to activate the YFP and the wavelength of an excitation laser beam 561 nm was used to activate the RFP. The wavelengths of the emission spectra of the YFP and the RFP were 522–552 nm and 565–615 nm, respectively. The images of spines, boutons, and synapse contacts were quantitatively analyzed by ImageJ and Imaris (Gao et al., 2019).

Before the electrophysiological recording of barrel cortical neurons, the mice in PSG, UPSG, and NCG were anesthetized by the intraperitoneal injections of urethane (1.5 g/kg) for surgical operations 5 days after the training paradigm was done. The body temperature was kept at 37°C by a computer-controlled heating blanket. The craniotomy (2 mm in diameter) was done on the mouse skull above the left side of the barrel cortex (−1.34 mm posterior to the bregma and 2.75 mm lateral to the midline), or a contralateral side of whisker stimulation (Wu et al., 2020). Electrophysiological recordings at barrel cortical neurons *in vivo* were conducted in these mice under a light anesthetic condition with the withdrawal reflex by pinching, the eyelid blinking reflex by air-puffing, and muscle relaxation. The unitary discharges of barrel cortical neurons in the category of local field potential (LFP) were recorded in layers II-III of the barrel cortices by using glass pipettes filled with a standard solution (150 mM NaCl, 3.5 mM KCl, and 5 mM HEPES). The resistance of the recording pipettes was 5–7 MΩ. The electrical signals of barrel cortical neurons in spontaneous spikes and evoked spikes by the whisker stimulus or the odorant stimulus were recorded and acquired by an AxoClamp-2B amplifier and a Digidata 1322A. The data were analyzed by pClamp 10 system (Axon Instrument Inc., Burlingame, CA, USA). Spiking signals were digitized at 20 kHz and filtered by a low pass at 5 kHz. A 100–3,000 Hz band-pass filter and the second-order Savitzky-Golay filter were used to isolate spike signals. The spike frequencies were quantitatively analyzed. The spike frequencies in response to the whisker stimulus and the odor stimulus were the ratio that the spike frequencies in response to the stimulations were divided by spontaneous spike frequencies 20 s before stimulations. If the ratio of evoked-spike frequencies to spontaneous spike frequencies was 1.2 or above, the barrel cortical neurons were deemed as responding to this stimulus (Wu et al., 2020).

In statistical analysis, the Mann–Whitney *U* test was used for the comparisons of experiment data before and after the associative learning as well as the neuronal responses to the whisker stimulation and the odorant stimulation in each of the mice. Two-way ANOVA was used for the statistical comparisons of alternations in neuronal activities and morphology among NCG, UPSG, and PSG mice.

## Results

### The strengthening of associative memory and sensitization

**FIGURE 1:**
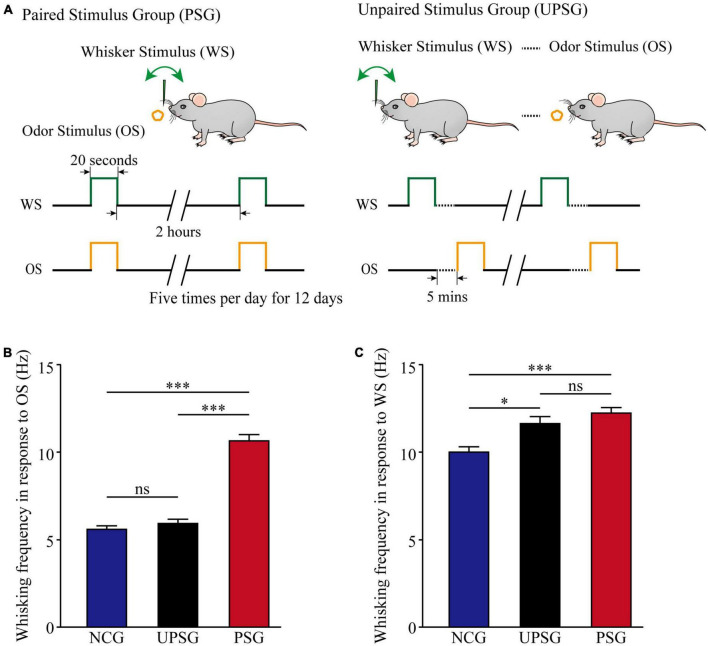
Whisking frequency is upregulated by the associative learning of whisker and odorant signals. The simultaneous pairings of the whisker stimulus (WS) and the olfactory stimulus (OS) lead to the emergence of odorant-induced whisker motion and the strengthening of whisking- induced whisker motion, in comparison with the sensitization of whiskers to the WS after the repeated WS. The WS was mechanical vibration pulses at 5 Hz, which was able to evoke whisker fluctuation. The OS was butyl acetate pulse to the noses, which sufficiently evoked the response of olfactory bulb neurons. The duration of both mechanical and odorant pulses were 20 s in the paired stimulus group (PSG) and the unpaired stimulus group (UPSG). **(A)** Left panel shows a paradigm in the pairing of the OS and the WS stimulus simultaneously to mice. The right panel shows the unpairing of the OS and the WS stimulus, in which the dashed line denotes an interval of about 5 min. **(B)** Shows whisking frequencies in response to the odor test in PSG mice (red bars, *n* = 14), in UPSG mice (black bars, *n* = 14), and in NCG mice (blue bars, *n* = 14). Three asterisks denote *p* < 0.001, and NS is no statistical significance (two-way ANOVA). **(C)** Shows whisking frequencies in response to the whisker test in PSG mice (red bars, *n* = 14), in UPSG mice (black bars, *n* = 14), and in NCG mice (blue bars, *n* = 14). One asterisk denotes *p* < 0.05, three asterisks show *p* < 0.001, and NS is no statistical significance (two-way ANOVA).

In comparison with mice in the naïve control group (NCG), the associative learning in C57BL/6J Thy1-YFP mice was conducted by the paired stimulations of the tactile vibration to mouse longer whiskers and the butyl acetate closely to the mouse noses for 12 days in the paired-stimulus group (PSG, left panel in Figure 1A), as well as non-associative learning, was done by the unpaired stimulations of the whisker tactile and the butyl acetate in unpaired-stimulus group (UPSG, right panel in Figure 1A; please also see Methods in detail). The formation of associative memory was accepted when the odorant-induced whisker motion emerged in the PSG mice. The upregulation of innate whisking-induced whisker motion in the PSG mice was thought of as the strengthening of associative memory. The upregulation of whisking-induced whisker motion in the UPSG mice was thought of as sensitization in the category of non-associative memory.

Figure 1B illustrates the comparison of whisker fluctuation (whisking) frequency in response to the olfactory stimulus (OS) among PSG, UPSG, and NCG mice. Whisking frequencies in response to the OS are 10.64 ± 0.33 Hz in PSG mice (red bar, *n* = 14), 5.96 ± 0.18 Hz in UPSG mice (black bar, *n* = 14), and 5.38 ± 0.18 Hz in NCG mice (blue bar, *n* = 14), respectively. *p*-values from the statistical comparisons of PSG mice versus NCG and UPSG mice are less than 0.001 (two-way ANOVA), but *p*-value for the comparison between NCG mice and UPSG mice equals 0.6. The emergence of the odorant-induced whisker motion in PSG mice indicates the formation of associative memory after associative learning by pairing the whisker stimulus and the olfactory stimulus.

Figure 1C shows the comparison of whisking frequencies in response to the whisker stimulus (WS) among PSG, UPSG, and NCG mice. Whisking frequencies in response to the whisker stimulus are 12.23 ± 0.29 Hz in PSG mice (red bar, *n* = 14), 11.63 ± 0.28 Hz in UPSG mice (black bar, *n* = 14), and 10 ± 0.37 Hz in NCG mice (blue bar, *n* = 14), respectively. The *p*-value from the statistical comparisons of NCG mice versus PSG mice is less than 0.001 (two-way ANOVA), the *p*-value from the comparison between NCG mice and UPSG mice is less than 0.05, as well as the *p*-value from the comparison between PSG mice and UPSG mice is 0.2. The upregulation of whisking-induced whisker motions in PSG mice indicates the strengthening of associative memory, and the upregulation of whisking-induced whisker fluctuations in UPSG mice indicates the sensitization in the category of non-associative memory. the studies about cellular mechanisms underlying the strengthening of associative memory and sensitization are presented below.

### The increase of spiking capacity in associative memory neurons

Activities of barrel cortical neurons were studied by *in vivo* recording their sequential action potentials, and their spike-encoding capability was quantified by meriting spiking frequency. The recruitment of associative memory cells from barrel cortical neurons are functionally defined as an increase of their spiking frequencies by about 20% in response to the OS alongside their innate responses to the WS. The upregulated refinement of associative memory cells is accepted when there is a significant increase in spiking frequency in response to the WS.

**FIGURE 2:**
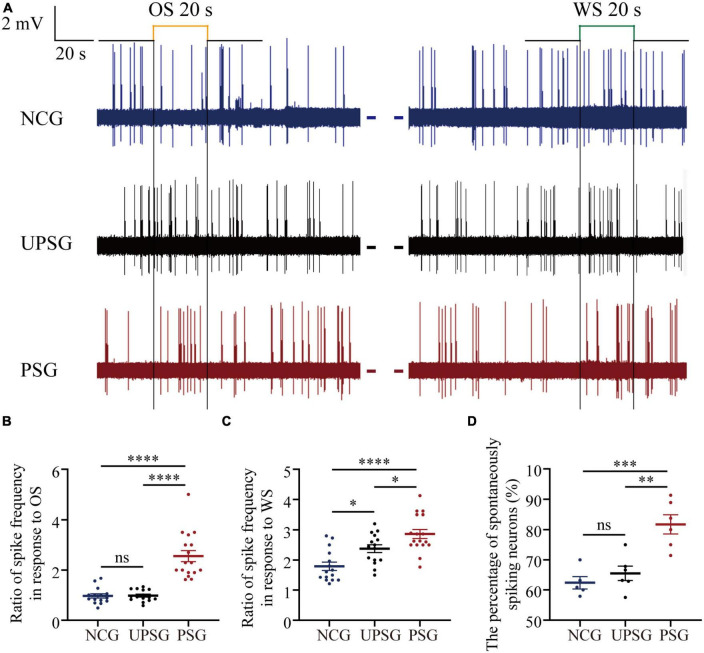
The spike-encoding capacity at barrel cortical neurons is upregulated by the associative learning of whisker and olfactory signals. Mice with associative memory show a higher spike encoding capacity in response to the WS and the OS on these barrel cortical neurons as well as a higher percentage of spontaneous-spiking neurons, compared to the other two groups, indicating the recruitment and upregulation of associative memory cells. Neuronal activities were recorded by unitary discharges in the category of local field potential (LFP). **(A)** Demonstrates LFPs recorded at barrel cortical neurons from PSG mice (red traces), UPSG mice (black traces), and NCG mice (blue traces) in response to the OS and the WS. Calibration bars are 2 mV and 20 s. **(B)** Shows the ratios of spike frequency in response to the OS from associative memory neurons in the barrel cortex in PSG mice (red symbols, *n* = 17 neurons from six mice) as well as from barrel cortical neurons in UPSG mice (black symbols, *n* = 15 neurons from five mice) and in NCG mice (blue symbols, *n* = 15 neurons from five mice). Four asterisks are *p* < 0.0001, and NS is no statistical significance (two-way ANOVA). **(C)** Shows the ratios of spike frequency in response to the WS from associative memory neurons in the barrel cortex in PSG mice (red symbols, *n* = 17 neurons from six mice), as well as from barrel cortical neurons in UPSG mice (black symbols, *n* = 15 neurons from five mice) and NCG mice (blue symbols, *n* = 15 neurons from five mice). Four asterisks demote *p* < 0.0001, and one asterisk is *p* < 0.05 (two-way ANOVA). **(D)** Shows the percentage of spontaneous spiking neurons in the barrel cortex from PSG mice (red symbols, data from six mice), UPSG mice (black symbols, data from six mice), and NCG mice (blue symbols, data from five mice). Three asterisks denote *p* = 0.0005, two asterisks are *p* = 0.0015 and NS is no statistical significance (two-way ANOVA).

Figure 2 illustrates electrical activities extracellularly recorded from barrel cortical neurons among mice in PSG, UPSG, and NCG. The spiking frequencies in response to the OS and/or the WS appear higher in barrel cortical neurons of PSG mice (red trace in Figure 2A) than in those of UPSG mice (black trace) and NCG mice (blue trace). Figure 2B illustrates that spiking frequencies in response to the OS are 2.56 ± 0.19 Hz in PSG mice (red symbols, *n* = 17 neurons from six mice), 0.98 ± 0.23 Hz in UPSG mice (black symbol, *n* = 15 neurons from five mice) and 0.97 ± 0.32 Hz in NCG mice (blue symbols, *n* = 15 neurons from five mice), respectively, in which four asterisks denote *p*-values less than 0.0001 (two-way ANOVA). What the barrel cortical neurons encode the new OS along with the innate WS in PSG mice implies their recruitment into associative memory neurons.

Moreover, Figure 2C shows that spike frequencies in response to the WS are 2.86 ± 0.62 Hz at associative memory neurons in PSG mice (red symbols, *n* = 17 neurons from six mice), 2.38 ± 0.5 Hz at barrel cortical neurons in UPSG mice (black symbols, *n* = 15 cells from five mice) and 1.79 ± 0.53 Hz at barrel cortical neurons in NCG mice (blue symbols, *n* = 15 cells from five mice), respectively, in which four asterisks denote *p*-values less than 0.0001 and one asterisk denotes *p*-value less than 0.05 (two-way ANOVA). The response of associative memory neurons to the WS in PSG mice is higher than the response of barrel cortical neurons in UPSG and NCG mice, indicating their functional upregulation to strengthen associative memory. Moreover, the response of barrel cortical neurons to the WS in UPSG mice being higher than in NCG mice indicates their functional upregulation for the sensitization. What the response of associative memory neurons to the WS in PSG mice being higher than that of barrel cortical neurons in UPSG mice indicates is the upregulated refinement of associative memory neurons.

Figure 2D illustrates that the percentage of associative memory neurons in the barrel cortex with spontaneous spikes is 81.73 ± 7.76% in PSG mice (red symbols, *n* = 6), in comparison with the percentages of barrel cortical neurons with spontaneous spikes at 65.51 ± 5.89% in UPSG mice (black symbols, *n* = 5) and 62.38 ± 4.66% in NCG mice (blue symbols, *n* = 5; three asterisks, *p* < 0.001; two asterisks, *p* < 0.01; two-way ANOVA). There are more associative memory neurons that show spontaneous activity in the barrel cortex from PSG mice than barrel cortical neurons that show spontaneous activity in UPSG and NCG mice, indicating the upregulated excitability of associative memory neurons after they are recruited from barrel cortical neurons. Compared to the cortical neurons, the increased spike-encoding capability at the associative memory neurons in response to cues and spontaneously is presumably driven by their interaction with the chemical synapses newly formed (Guo et al., 2017; Zhao et al., 2017), which was examined morphologically.

### The increased interconnections among associative memory neurons in the barrel cortex

**FIGURE 3:**
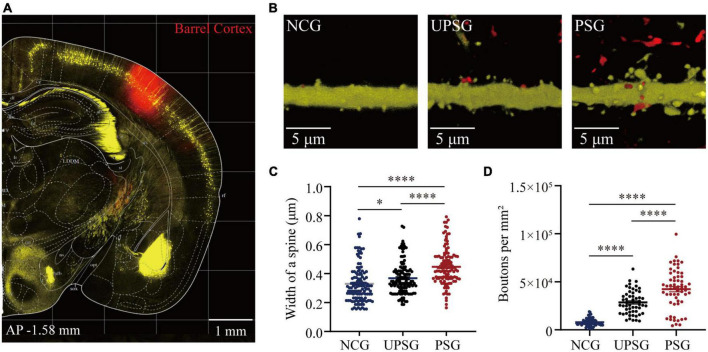
Barrel cortical neurons in PSG mice that experience the associative learning of whisker signal and olfactory signal show an increase in the width of one spine and the number of boutons compared to UPSG mice and NCG mice. **(A)** Shows RFP expression at the AAV-CMV-tdTomato injection site in the barrel cortex for neural tracing within the local area. Calibration bar is 1 mm. **(B)** Shows RFP-labeled axonal boutons and YFP-labeled post-synaptic dendrites in the barrel cortex from NCG mice (left panel), UPSG mice (middle panel), and PSG mice (right panel). Calibration bar is 5 μm. **(C)** Shows the width of spines on barrel cortical neurons in PSG mice (red dots, *n* = 125 dendrites from five mice), UPSG mice (black dots, *n* = 125 dendrites from five mice), and NCG mice (blue dots, *n* = 125 dendrites from four mice). One asterisk is *p* = 0.0304, and four asterisks are *p* < 0.0001 (two-way ANOVA). **(D)** Shows axonal boutons per mm^2^ in barrel cortices from PSG mice (red dots, *n* = 59 confocal images from five mice), UPSG mice (black dots, *n* = 53 confocal images from four mice), and NCG mice (blue dots, *n* = 73 confocal images from five mice). Four asterisks denote *p* < 0.0001 (two-way ANOVA).

If the interconnections rise among associative memory neurons and/or cortical neurons, we expect to see the increase in synapse contacts between presynaptic boutons and post-synaptic spines. In the meantime, the presynaptic boutons and post-synaptic dendritic spines may increase in their densities. To study the interconnections and their upregulated refinements of associative memory neurons and cortical neurons within the barrel cortex, we have microinjected AAV-CMV- tdTomato into the barrel cortex in PSG, UPSG, and NCG mice. The tdTomato-carried viruses were uptaken into and expressed in associative memory neurons in PSG mice as well as barrel cortical neurons in UPSG and NCG mice (Figure 3A). The RFP was transported in an anterograde manner into entire presynaptic axonal branches including boutons and terminals. YFP-labeled pyramidal neurons genetically in the barrel cortex were their post-synaptic targets. The contacts between RFP-labeled boutons and YFP-labeled spines are thought of as the synapse contacts within the intramodal cortex by this approach.

Figures 3B–D shows the width of spines at YFP-labeled pyramidal neurons and the densities of axonal boutons in barrel cortices from the PSG, UPSG, and NCG mice, respectively, in which these parameters appear higher toward lower from PSG mice, UPSG mice to NCG mice (Figure 3B). The widths of dendritic spine heads on barrel cortical neurons are 0.45 ± 0.13 μm in PSG mice (red symbols in Figure 3C, *n* = 125 dendrites from five mice), 0.37 ± 0.11 μm in UPSG mice (black symbols, *n* = 125 dendrites from five mice) and 0.33 ± 0.13 μm in NCG mice (blue symbols, *n* = 125 dendrites from four mice), in which four asterisks show *p* < 0.0001 and one asterisk is *p* < 0.05 (two-way ANOVA). Therefore, the width of spine heads on barrel cortical neurons in PSG mice is higher significantly than those in UPSG mice, and the width of spine heads on barrel cortical neurons in UPSG mice is significantly higher than those in NCG mice. Such data indicate that the diameter of dendritic spines is upregulated in PSG mice for the refinement of associative memory neurons and in UPSG mice for the sensitization of barrel cortical neurons.

Moreover, the densities of axonal boutons per mm^2^ that were calculated from the confocal images under 60X within the barrel cortices from PSG mice are 4.23 × 10^4^ ± 2.09 × 10^4^ per mm^2^ (red symbols in Figure 3D, *n* = 59 confocal images from five mice), 2.85 × 10^4^ ± 1.25 × 10^4^ per mm^2^ in UPSG mice (black symbols, *n* = 53 confocal images from four mice) and 0.79 × 10^4^ ± 0.37 × 10^4^ per mm^2^ in NCG mice (blue symbols, *n* = 73 confocal images from five mice), in which four asterisks denote *p* < 0.0001 (two-way ANOVA). Therefore, the densities of pre-synaptic axonal boutons from barrel cortical neurons in PSG mice are significantly higher than those in UPSG mice, and the densities of pre-synaptic axonal boutons in UPSG mice are significantly higher than those in NCG mice. The data indicate that the number of pre-synaptic axon boutons has been upregulated in PSG mice for the refinement of associative memory neurons as well as in UPSG mice for the sensitization of barrel cortical neurons.

**FIGURE 4:**
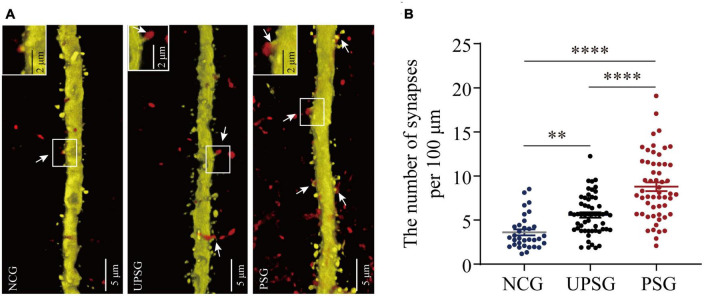
Barrel cortical neurons in PSG mice with the associative learning of whisker signal and olfactory signal show an increase in the number of synapses, compared with UPSG and NCG mice. **(A)** Shows the synapse contacts between RFP-labeled axon boutons and YFP-labeled post-synaptic dendritic spines in barrel cortices from NCG mice (left panel), UPSG mice (middle panel), and PSG mice (right panel). White arrows indicate these contacts. Left-top frames in each of the panels show the enlarged synapse contacts from the white boxes. Calibration bars are 5 μm in each of the panels, and calibration bars in white enlarged frames are 2 μm. **(B)** Shows synapses per 100 μm dendrites in barrel cortices from PSG mice (red dots, *n* = 55 dendrites from five mice), UPSG mice (black dots, *n* = 55 dendrites from five mice), and NCG mice (blue dots, *n* = 33 dendrites from four mice). Two asterisks are *p* = 0.0057, and four asterisks are *p* < 0.0001 (two-way ANOVA).

In the analysis of the synapse contacts between pre-synaptic axon boutons and post-synaptic spines in Figure 4, we also observed that the densities of synapse contacts appear higher toward lower from PSG mice, UPSG mice to NCG mice (Figure 4A). The densities of synapse contacts per 100 μm dendrites in barrel cortices are 8.79 ± 3.72 per 100 μm from PSG mice (red symbols in Figure 4B, *n* = 55 dendrites from five mice), 5.58 ± 2.25 per 100 μm in UPSG mice (black symbols, *n* = 55 dendrites from five mice) and 3.63 ± 1.93 per 100 μm in NCG mice (blue symbols, *n* = 33 dendrites from four mice), in which four asterisks show *p* < 0.0001 (two-way ANOVA) and two asterisks are *p* < 0.01. Thus, the synapse contacts on cortical neurons in PSG mice are significantly higher than those in UPSG mice, and the synapse contacts in UPSG mice are significantly higher than those in NCG mice. These results indicate that the synapse contacts are upregulated in PSG mice for the refinement of associative memory neurons and in UPSG mice for the sensitization of barrel cortical neurons.

## Discussion

By giving paired whisker tactile and olfaction signals to mice for associative learning, we have investigated the formation and strengthening of associative memory as well as their cellular mechanisms with functional and morphological approaches. The emergence of odorant-induced whisker motion and the upregulation of whisking-induced whisker motion (Figure 1) indicates the strengthening of association memory along with the formation of associative memory. In the meantime, barrel cortical neurons in the mice experiencing associative learning become able to encode the olfactory signal alongside the innate whisker signal, or the recruitment of associative memory neurons for the formation of associative memory. These associative memory cells show the increase of their spike-encoding capability in response to input signals, i.e., the strengthening and consolidation of associative memory (Figure 2). The upregulation of associative memory neurons appears based on the rises in post-synaptic dendritic spines, pre-synaptic axonal boutons, and synapse contacts (Figures 3, 4).

In terms of neuronal correlates underlying the formation of associative memory, our studies have indicated the recruitment of associative memory cells in cerebral cortices, which is featured by the mutual synapse innervations among cross-modal cortices along with their innate synapse innervation (Wang et al., 2015, 2018, 2019; Gao et al., 2016, 2019; Yan et al., 2016; Feng et al., 2017; Lei et al., 2017; Liu et al., 2017; Wu et al., 2020). In this study, we further present that such associative memory neurons within the intramodal cortex are upregulated in their spike-encoding capability and their recurrent synapse interconnections *in vivo* (Figures 2–4). Theoretically, the decreases in the threshold and refractory period of action potentials, as well as the increase of synapse-driving force, can strengthen neuron activities in spike encodings. The morphological upregulation of synapse interconnections among associative memory neurons (Figures 3, 4) should enhance their spike-encoding ability. Moreover, the conversion of silent synapses into functional synapses (Liao et al., 1995) and the conversion of inactive synapses into active synapses (Wang and Kelly, 2001) increase the synapse-driving force to strengthen the activities of associative memory neurons (Guo et al., 2017; Zhao et al., 2017; Wang, 2019). Taken together, our studies support the hypothesis about the activity-dependent positive recycle in the recruitment and refinement of associative memory neurons for the formation and consolidation of associative memory (Wang, 2019).

In the pairing of the whisker tactile stimulus signal and the odorant stimulus signal for their associative learning, barrel cortical neurons and piriform cortical neurons are coactivated by their specific innate inputs (Gao et al., 2016, 2019; Feng et al., 2017; Lei et al., 2017; Wu et al., 2020). The intensive activities of these cortical neurons driven by sequential action potentials and of their synapses via ionotropic and metabotropic receptors instigate intracellular epigenetic events and subsequent epigenetic-regulated expression of some genes and proteins in these neurons including NET3, neuroligin-3, and ttbk1. The axons of the barrel and piriform cortical neurons are projected toward mutual directions, and their synapse interconnections are formed to recruit associative memory cells among cross-modal cortices for the formation of associative memory (Feng et al., 2017; Lei et al., 2017; Wang et al., 2019; Wu et al., 2020). Moreover, the coactivity of intramodal cortical neurons (such as barrel cortical neurons in the present study), which are driven by the synapse innervation from the piriform cortex as well as the synapse input from the innate whisker signal, can trigger the recurrent synapse innervations mutually among associative memory cells within the barrel cortex through this activity-induced epigenetic process. In addition to coactivity-dependent synapse formation for the recruitment of associative memory cells, the coactivity of associative memory neurons may activate intracellular Ca2 + /calmodulin signaling pathway for the conversion of inactive synapses into active synapses (Wang and Kelly, 2001; Wang et al., 2008) and the upregulation of neuron-encoding capacity (Zhang et al., 2004; Chen et al., 2008) for the strengthening and consolidation of associative memory. Therefore, our study grants two ideas, or the activity together and the interconnection together (Wang, 2019) as well as the activity together and the strengthening together (Hebb, 1949).

Neural substrates for information storage have been originally termed memory traces or engrams, which have been presumably thought of as the biophysical and biochemical changes in the brain (Simon, 1921, 1923), the strengthened interconnection of cell assemblies (Hebb, 1949), the synaptic plasticity (Bliss and Lynch, 1988; Govindarajan et al., 2006; Poo et al., 2016) and the neuronal plasticity (Armano et al., 2000; Zhang et al., 2004) in response to the learning and training. The characteristics of cell assemblies in memory traces or engrams remain far away to be known. Engrams have been found to be labeled by immediate early genes as their molecular markers (Josselyn et al., 2015; Tonegawa et al., 2015; Pignatelli et al., 2018). These molecules indicate the strength of neuron activities, but not the specific correlation for a group of memory neurons to encode definite associative signals (Wang, 2019). We have paid attention to investigating the formation and features of basic units in memory traces or engrams. As the most common style of learning and memory is associative in nature about multiple signals, i.e., their joint storages and reciprocal retrievals, we expect to see those basic units in engrams or memory traces that can encode these associated signals, which have not been reported previously. Our systemic studies have successfully identified certain brain cells being recruited to encode multiple signals from associative learning (Wang et al., 2015, 2018, 2019; Gao et al., 2016, 2019; Yan et al., 2016; Feng et al., 2017; Lei et al., 2017; Liu et al., 2017; Wu et al., 2020). These basic units have been called associative memory cells since they are morphologically interconnected and functionally encode multiple associated signals after the memories of these signals arise (Wang et al., 2015; Feng et al., 2017). Associative memory cells possess the following characteristics (Wang, 2019). The associative memory cells are recruited from the coactivity of cortical neurons. The simultaneous coactivity of cortical neurons causes their interconnections. That is, a group of neurons receives new synapse innervations from other groups of neurons ever being coactive besides their innate synapse inputs. These cortical neurons become able to encode associative signals inputted from new and innate synapse innervations (Wang, 2019). Because these cortical neurons receive synapse innervations from multiple sources and encode their inputted signals persistently, their functions show the association of specific learned signals and cues for memories, such that these cortical neurons are figuratively termed to be associative memory neurons or cells, instead of engram cells that have not been represented as the features of neural substrates for basic units to encode and memorize multiple signals associated during the learning (Wang, 2019). In this regard, we would suggest examining whether the neurons labeled by activity-expressed genes (e.g., immediate early genes) are able to encode all signals and cues acquired during the associative learning in the long-term as well as to receive synapse innervations of inputting these signals from those studies in the society for memoriology.

**FIGURE 5:**
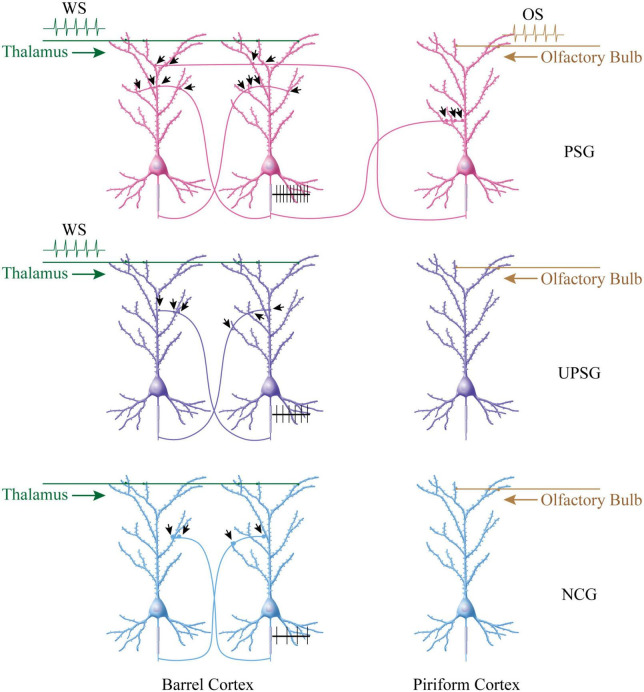
More new synapse innervations among associative memory cells in the barrel cortex are recruited by the associative learning of whisker and olfactory signals in PSG mice, compared to barrel cortical neurons in UPSG mice and NCG mice. **(Top panel)** Shows that more new synapse interconnections are formed among associative memory neurons in the barrel cortex from PSG mice. The simultaneous pairings of the whisker signal from the thalamus and the odorant signal from the olfactory bulb evoke the coactivity of barrel cortical and piriform cortical neurons, which drives mutual synapse innervations between barrel cortical neurons and piriform cortical neurons to recruit associative memory neurons. The innate synapse input from the thalamus and the new synapse input from the piriform cortex drive barrel cortical neurons to be coactive, leading to the formation of numerous new synapses among associative memory cells within the barrel cortex. These synapses may drive the strong activity of these associative memory neurons (black trace showing their sequential spikes). **(Middle panel)** Illustrates the moderate increase of new synapses among barrel cortical neurons from UPSG mice, which are induced by the whisker signal from the thalamus without the synapse driving force from the piriform cortex. **(Bottom panel)** Shows a few synapse innervations among barrel cortical neurons from NCG mice since these neurons have not received intensive synapse driving force from the thalamus and/or the piriform cortex. The synapse interconnections among barrel cortical neurons as well as their spike-encoding capacity are influenced by the quantity of synapse-input sources.

It is noteworthy that the upregulations in the interconnection and the interaction among the intramodal cortical neurons can be induced by their intensive activities driven by innate synapse inputs. For instance, the spike-encoding ability and the recurrent synapse innervation rise among barrel cortical neurons significantly in UPSG mice, in comparison with NCG mice and PSG mice (Figures 2–4). These data indicate that intensive activities at barrel cortical neurons by their innate inputs sufficiently strengthen interconnections and interactions among those intramodal cortical neurons for the sensitization of non-associative learning in a single neural pathway, which are the supplement mechanisms for non-associative learning and memory via the long-term potentiation of synaptic transmission and neuronal spike-encoding in a single pathway previously reported (Bliss and Lomo, 1973; Bliss and Lynch, 1988; Wang et al., 1997; Armano et al., 2000; Zhang et al., 2004; Poo et al., 2016; Lisman et al., 2018). The advanced upregulations of synapse interconnections, neuron interactions, and even neuron spontaneous activities among associative memory neurons recruited from barrel cortical neurons indicate that the new synapse innervations projected from cross-modal cortices can drive and induce these alternations, which are additive onto the driven force from the innate synapse inputs. Thus, both innate inputs and new inputs can induce similar changes, reinforcing a rule, the activity together, the connection together, and the strengthening together, for positive cycling in the recruitment and refinement of memory cells with distinct strengths (Figure 5).

Neural tracing has been broadly used to address the connection among different cortical areas (Wang, 2019). In the present study, we have injected AAV-carried genes for fluorescent proteins in a cortical area as well as detected axon boutons and their targets in this injection area. After AAV-carried genes are uptaken by cortical neurons and fluorescent proteins are expressed in these cortical neurons, the anterograde transportation of fluorescent proteins along with their axons in the local area allows us to examine axonal innervations and neuronal interconnections regionally. By using this approach, we have demonstrated the morphological evidence about the interconnection and interaction of associative memory cells within the intramodal cortex for the strengthening of associative memory. Our study also grants the activity-dependent refinement of associative memory cells by the growth of post-synaptic dendrites and presynaptic axons for the formation of *en passant* synapses (Figure 5; Wang, 2019). Our approach and results also provide morphological evidence for the interconnection of pair-encoding neurons within the visual cortex in vision-relevant associative memory (Sakai and Miyashita, 1991; Naya et al., 2003; Albright, 2012).

## Data availability statement

The original contributions presented in this study are included in the article/supplementary material, further inquiries can be directed to the corresponding author.

## Ethics statement

The animal study was reviewed and approved by the Institutional Animal Care and Use Committee in the Administration Office of Laboratory Animal at Beijing China (B10831).

## Author contributions

J-YL, YX, and D-GW contributed to the experiments and data analyses. J-HW contributed to the concept, project design, and manuscript writing. All authors contributed to the article and approved the submitted version.
